# Novel Microbial Enzymes with Industrial Applications

**DOI:** 10.3390/microorganisms11040986

**Published:** 2023-04-10

**Authors:** María-Isabel González-Siso, Manuel Becerra

**Affiliations:** CICA—Centro Interdisciplinar de Química e Bioloxía, Facultade de Ciencias, Universidade da Coruña, 15071 A Coruña, Spain

Eberhardt et al. [1] achieved the identification of novel genes encoding β-galactosidases by means of sequence-based metagenomics of dairy industry stabilization ponds for wastewater treatment, into which lactose-rich effluents are poured. Several hundred bacterial genes were annotated as β-galactosidases, among which a group were identified as being expressed in *Escherichia coli* and characterized, and one of these showed remarkable activity, being very promising for the synthesis of value-added products, such as lactose hydrolysates or galactooligosaccharides, from dairy industry effluents. Kamli et al. [2] performed comprehensive comparative analyses of the genomes of nine species of the under-studied genus *Aneurinibacillus*. Antimicrobial and antiviral activities, carbohydrate-active enzymes and genes involved in heavy metal resistance were identified. The potential of this bacterial genus for several industrial applications was revealed. Guo et al. [3] isolated bacterial strains from the sediment of deep-sea cold seep, finding a novel strain that exhibited high cold-active (4 °C) lipase activity. This strain was proposed to constitute a new species of *Pseudomonas*, and was named *P. marinensis*. Two genes encoding for cold-active lipases were identified in its genome, although one lipase showed much higher activity and stability than the other. This difference was explained in the function of the aminoacidic composition. Through mutagenesis experiments, the Ser/Asp/His catalytic triads of both lipases were proved essential for enzyme activity. These enzymes are promising for several industries due to the reduced energy consumption required for the catalysis. Silva-Salinas et al. [4] studied the α-amylase activity produced by *Bacillus licheniformis* strain LB04, isolated from hot springs in Mexico. The enzyme was immobilized by entrapment on agar-agarose beads showing a higher hydrolytic activity than the free enzyme. It was stable at pH 3.0 and up to 80 °C, and was found to be efficient for starch hydrolysis at temperatures above 65 °C and in acidic conditions, making this α-amylase interesting for the refined syrup and bakery industries. Almahasheer et al. [5] isolated five keratinolytic bacteria (identified as members of the *Bacillus cereus* group) from poultry farm waste in Saudi Arabia. Keratinase activity was enhanced through random mutagenesis. Mutations did not affect the subtilisin-like serine protease domain containing the Asp/His/Ser catalytic triad. Due to their thermostability, activity at alkaline pH and binding affinity, the keratinases are potentially useful for industrial feather processing. Wang et al. [6] compared the activities of the cellulase systems of the thermophilic anaerobic bacteria *Acetivibrio thermocellus* and *Thermoclostridium stercorarium* using several kinds of hemicellulose and cellulose as substrates. *A. thermocellus* mainly performed cellulose hydrolysis, whereas *T. stercorarium* mainly performed hemicellulose hydrolysis. The two cellulase systems proved to have a synergistic effect. Ethanol yields of the co-culture of *A. thermocellus* and *T. stercorarium* nearly doubled those of the monocultures, which indicates a novel method of ethanol production from cellulosic biomass through consolidated bioprocessing. González-González et al. [7] tested KLEST-3S esterase (the thermoalkalophilic membrane-associated esterase from *Thermus thermophilus* HB27 cloned and expressed in *Kluyveromyces lactis*) as a biocatalyst in aqueous and organic media. The recombinant enzyme showed high thermal stability and activity in the presence of 10% (*v*/*v*) organic solvents and 1% (*w*/*v*) detergents. KLEST-3S provided high yields for the acetylation of alcohols, and catalyzed the stereoselective hydrolysis of (*R*,*S*)-ibuprofen methyl ester (87% ee). It is potentially applicable for industrial bioconversions.

These published articles represent new and exciting contributions to upgrade enzyme biotechnology-based industries.
